# Olfactory discrimination of anal sac secretions in the domestic cat and the chemical profiles of the volatile compounds

**DOI:** 10.1007/s10164-017-0532-x

**Published:** 2017-11-16

**Authors:** Tamako Miyazaki, Takashi Nishimura, Tetsuro Yamashita, Masao Miyazaki

**Affiliations:** Department of Biological Chemistry and Food Sciences, Faculty of Agriculture, Iwate University, 3-18-8 Ueda, Morioka, Iwate 020-8550 Japan

**Keywords:** Scent communication, Chemical signals, Volatile compounds, Individual recognition, Free fatty acids

## Abstract

Scent emitted from anal sac secretions provides important signals for most Carnivora. Their secretions emit a variety of volatile compounds, some of which function as chemical signals with information about the scent owners. The domestic cat has a pair of anal sac glands to secrete a pungent liquid. Their anal sac secretions may give information about sex, reproductive state, and recognition of individuals. However, little is known about the volatile compounds emitted from anal sac secretions and their biological functions in cats. In this study, we examined the volatile chemical profiles of anal sac secretions in cats and their olfactory ability to discriminate intraspecific anal sac secretions. Analysis with gas chromatography–mass spectrometry showed that the major volatile compounds were short-chain free fatty acids, whose contents varied among individuals, as well as other carnivores. There was no sex difference in the volatile profiles. In temporal analyses of individual anal sac secretions performed 2 months apart, the profiles were highly conserved within individuals. Habituation–dishabituation tests showed that cats can distinguish individual differences in the odor of anal sac secretions. These results suggest that cats utilize short-chain free fatty acids emitted from anal sac secretions to obtain scent information for individual recognition rather than species or sex recognition.

## Introduction

Most Carnivora have a pair of anal sacs that contain the secretions of the anal glands and open to each side of the anus (Gorman and Trowbridge 1989). Anal sac secretions contain volatile compounds that emit strong pungent odors used for scent communication in mammals. Anal sac secretions are used for defense by the skunk (*Mephitis macroura*) (Wood et al. 2002) and the honey badger (*Mellivora capensis*) (Begg et al. 2003), territory marking by the hyena (*Crocuta crocuts*) (Drea et al. 2002) and the wolf (*Canis lupus*) (Asa et al. 1985), individual identification by the ferret (*Mustela furo* Linnaeus) (Clapperton et al. 1988), the mongoose (*Herpestes auropunctatus*) (Gorman 1976), the giant panda (*Ailuropoda melanoleuca*) (Zhang et al. 2008), and the spotted hyena (*Crocuta crocuta*) (Burgener et al. 2009), and sex recognition by the brown bear (*Ursus arctos*) (Rosell et al. 2011), the giant panda (*Ailuropoda melanoleuca*) (Yuan et al. 2004), and some mustelids (*Mustela* spp.) (Zhang et al. 2002, 2005). Chemical analyses of anal sac secretions of the red fox (*Vulpes vulpes*), dog (*Canis familiaris*), coyote (*Canis latrans*), wolf (*Canis lupus*), and mongoose have identified volatile short-chain free fatty acids, such as acetic acid, propanoic acid, and butanoic acid as being responsible for the odors (Albone and Perry 1976; Apps et al. 2012; Decker et al. 1992; Preti et al. 1976; Raymer et al. 1985).

The domestic cat has a pair of anal sacs lined with glands of two types, apocrine and sebaceous, within the ventrolateral perianal region (Shoieb and Hanshaw 2009). It has been reported that anal sacs release a pungent liquid secretion into a cat’s feces for territorial marking, and such secretions may have information about sex, reproductive state, and recognition of individuals (Feldman 1994). Surprisingly, however, there is little scientific evidence of the contribution of anal sac secretions to scent communication in cats. Most studies of anal sacs have focused on disorders, including carcinoma (Mellanby et al. 2002; Parry 2006; Shoieb and Hanshaw 2009) and gross and cytological characteristics for diagnostic purposes (Frankel et al. 2008). Although millions of cats coexist with humans worldwide, the scientific community is only beginning to understand the cognition and behavior of cats (Vitale Shreve and Udell 2015). Considering that chemical signals emitted from their excretions, secretions, and bodies are important for scent communication in cats (Shreve and Udell 2017), it is necessary to examine the function and chemical profile of anal sac secretions in them. Such studies will improve our understanding of behavior and scent communication in domestic cats, and also may help to address behavior issues, as reported in our recent study (Miyazaki et al. 2017a).

In this study, we first analyzed the headspace gas of the anal sac secretions for chemical profiling of the volatile compounds emitted from the secretions by using a thermal desorption gas chromatography–mass spectrometry (GC–MS) system. Then we carried out multivariate analysis using the data to examine sex and individual differences between the secretions. To test whether there are fixed patterns in volatile chemical profiles of anal sac secretions in each cat, we also analyzed the headspace gas of the secretions that were obtained in three cats at two points in time (day 1 and day 72). Finally, we examined whether cats can distinguish differences in anal sac secretions by their olfaction. To the best of our knowledge, this is the first study to examine anal sac secretions by domestic cats using both analytical and behavioral techniques.

## Materials and methods

### Animals and sample collection

Anal sac secretions were collected from 13 mixed breed cats (2–4 years old) including 8 intact males (identification numbers: ID. M1–M8) and 5 females (ID. F1–F5). Anal sac secretions of five male cats (M1–M5) and five female cats (F1–F5) were obtained only once as described below. Anal sac secretions of three male cats (M6–M8) were obtained twice at two points in time (day 1 and day 72). Five male cats (M1–M5) were also used for behavioral bioassays as described below. We selected the five males for the bioassays because they had a mild disposition and we could easily handle them. The cats were housed individually in cages that were kept at 22 °C under 12-h light: 12-h dark conditions. The Animal Research Committee of the Faculty of Agriculture, Iwate University approved the protocols for sample collection and all behavioral experiments using laboratory cats.

Anal sac secretions were obtained by squeezing each pair of sacs by hand with gloves (LAVENDER NITRILE Powder-Free Exam Gloves, Kimberly-Clark, Roswell, GA, USA). Since the secretions which were released through the small openings on either side of the anus stuck to the gloves, we transferred the secretions into a 20-ml glass vial (20 ml vial with screw neck, GESTEL, Mülheim an der Ruhr, Germany) using a spatula, and then closed the cap (screw cap, septum: silicone blue/PTFE white, 45° Shore A, 1.3 mm, GESTEL) for later analyses.

### Gas chromatography–mass spectrometry (GC–MS) analysis of headspace gas from anal sac secretions

Fresh samples of anal sac secretions (50-mg aliquot) were analyzed within 1 h after obtaining them from the 13 cats (M1–M8 and F1–F5). Anal sac secretions from the same individuals were obtained in three male cats (M6–M8) at two points in time (day 1 and day 72) and examined to see temporal changes in volatile chemical profiles of anal sac secretions. Each anal sac secretion sample was introduced into a new 20-ml glass vial. After closing the cap, the headspace gas above the sample was concentrated into an adsorption glass tube containing 300 mg of Tenax TA (Shimadzu, Kyoto, Japan) at 40 °C by purging with pure nitrogen gas at 50 ml/min for 60 min. Volatile compounds trapped in the Tenax TA were desorbed at 250 °C for 10 min using a thermal desorption system (TD-20; Shimadzu, Tokyo, Japan), and were then injected directly into a QP-2010 Ultra GC–MS equipped with a Stabilwax capillary column (length, 60 m; internal diameter, 0.32 mm; layer thickness, 0.5 µm; Restek, Bellefonte, PA, USA). GC was performed in splitless mode. The oven temperature was held at 40 °C for 2 min, increased to 250 °C at 8 °C/min, and held at 250 °C for 20 min. The mass spectrometer was operated in electron impact mode at an electron energy of 70 eV and ion source temperature of 200 °C. Mass spectra were obtained in full-scan mode from *m*/*z* 35 to *m*/*z* 300. The molecular species of the volatile compounds were identified by comparing the mass spectra and GC retention time indices with National Institute of Standards and Technology (NIST) library data and synthetic standards. The amounts of the 10 free fatty acids emitted from 50 mg anal sac secretions by purging with pure nitrogen gas at 50 ml/min for 60 min at 40 °C were quantified by the area obtained from the reprocessed chromatogram using the characteristic *m*/*z* fragments. Artificial synthetic free fatty acids were used to generate a standard curve. Acetic acid (purity > 99.5%), propanoic acid (purity > 98.0%), 2-methylpropanoic acid (purity > 99.0%), butanoic acid (purity > 99.0%), 3-methylbutanoic acid (purity > 99.0%), pentanoic acid (purity > 98.0%), 3-methylpentanoic acid (purity > 98.0%), 4-methylpentanoic acid (purity > 97.0%), and hexanoic acid (purity > 98.0%) were purchased from Tokyo Chemical Industry Co. (Tokyo, Japan). 4-Methylhexanoic acid (purity > 97%) was purchased from Sigma-Aldrich (St. Louis, USA). Total sums of the free fatty acids emitted from the samples were compared between males and females using the Wilcoxon rank sum test. Results were considered significant at *p* < 0.05.

GC–MS Solution software (ver. 4.20) was used to process the raw instrument data, including selecting peaks from the total ion chromatogram (TIC). All peak lists were further analyzed using JMP software (ver. 12.0) for principal component analysis (PCA) and hierarchical clustering analysis (HCA).

### Behavioral bioassays in cats

The habituation-dishabituation test was conducted in five male cats (M1–M5) using anal sac secretions obtained from two other male cats (M6 and M7). Anal sac secretions (aliquot, 50 mg) were placed in the bottom of a 20-ml glass vial closed by a screw cap without a septum immediately before the bioassays, which were conducted in a 54 × 74 × 60-cm test chamber. Each cat was introduced into the chamber 5 min before beginning the assay. In preliminary experiments that examined only their responses toward sequential presentations of anal sac secretions, the anal sac secretion obtained from a male cat (M8) was presented to the five cats. In habituation-dishabituation tests, an anal sac secretion sample of a male cat (M6) was presented twice sequentially in the first and second assays for 60 s at 30-s intervals; then, the other anal sac secretion sample of a male cat (M7) was presented once in the third assay for 60 s. In all experiments, the behavior of each cat was video-recorded using a digital video camera (Handycam HDR-CX560 V; Sony, Tokyo, Japan). Sniffing duration was counted when cats brought their noses to the tube openings and twitched their noses as they sniffed to sample the odors. Statistical significance was tested using repeated-measures analysis of variance, followed by Tukey’s honestly significant difference test, in JMP software (ver. 12.0; SAS Institute, Cary, NC, USA).

## Results

### Chemical profiles of anal sac secretions of cats

**Fig. 1:**
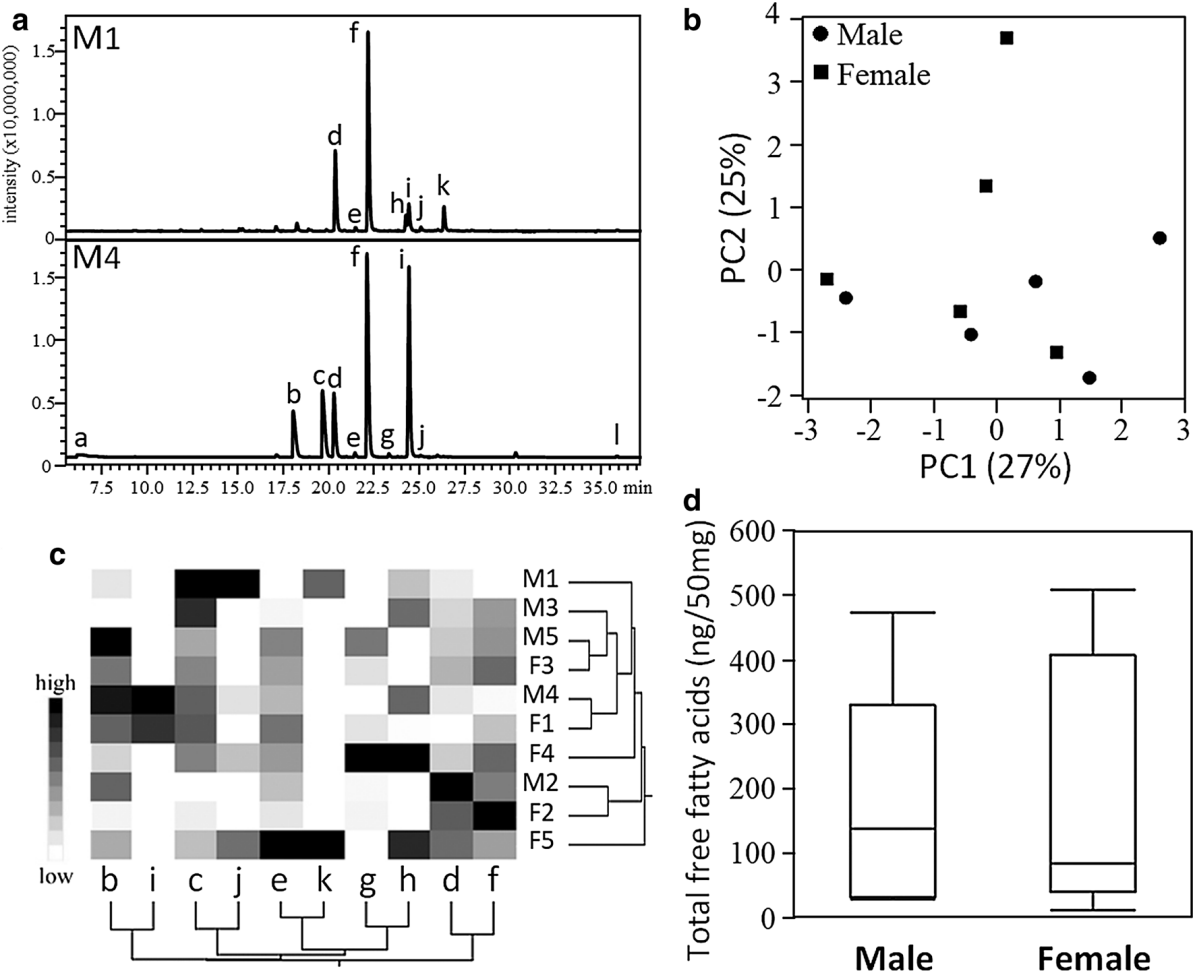
Chemical profiles of volatile compounds emitted from anal sac secretions of domestic cats **a** Representative GC–MS TICs of volatile compounds emitted from anal sac secretions of male cats. M1 and M4 mean identification numbers of two male cats. *a* Trimethylamine, *b* acetic acid, *c* propanoic acid, *d* 2-methylpropanoic acid, *e* butanoic acid, *f* 3-methylbutanoic acid, *g* pentanoic acid, *h* 3-methyl-pentanoic acid, *i* 4-methylpentanoic acid, *j* hexanoic acid, *k* 4-methylhexanoic acid, *l* indole. *X*-axis indicates retention time (min) and *y*-axis indicates intensity. **b** PCA was carried out using GC–MS data of the identified 10 free fatty acids emitted from five male and five female anal sac secretions. **c** Heat map and dendrograms were produced from GC–MS data of the 10 free fatty acids emitted from anal sac secretions of five male (M1–M5) and five female (F1–F5) cats. Ward’s minimum variance was used for hierarchical clustering. **d** Box and whisker plot of the total sum of amounts (ng) of the 10 free fatty acids emitted from 50-mg anal sac secretions of the five male (M1–M5) and five female (F1–F5) cats by purging with nitrogen gas at 50 ml/min for 60 min at 40 °C

Figure 1a shows a representative GC–MS total ion chromatogram of the volatile compounds emitted from the anal sac secretions of eight male and five female cats. The major volatile compounds in both sexes were short-chain free fatty acids, including acetic acid, propanoic acid, 2-methylpropanoic acid, butanoic acid, 3-methylbutanoic acid, and pentanoic acid. Trimethylamine and indole were minor components in the anal sac secretions. Since the chemical profiles of the 10 free fatty acids varied among individuals, the GC–MS data from the five male and five female cats were subjected to PCA, an unsupervised technique for reducing dimensionality, and HCA, which is used to group samples based on their similarity and thus construct a hierarchy of groups. PCA, in which the first two principal components together explained 52% of the total variance in the chemical profiles, and HCA showed that the major driver of variation among the samples was individual differences, and not sex differences (Fig. 1b, c). Figure 1d shows the total amount of free fatty acids emitted from 50 mg anal sac secretion samples of intact male and female cats, obtained by purging with nitrogen gas (50 ml/min) for 60 min. There was no difference in the total amount of free fatty acids between sexes.

### Volatile chemical profiles of anal sac secretions obtained on different days in same cats

**Fig. 2:**
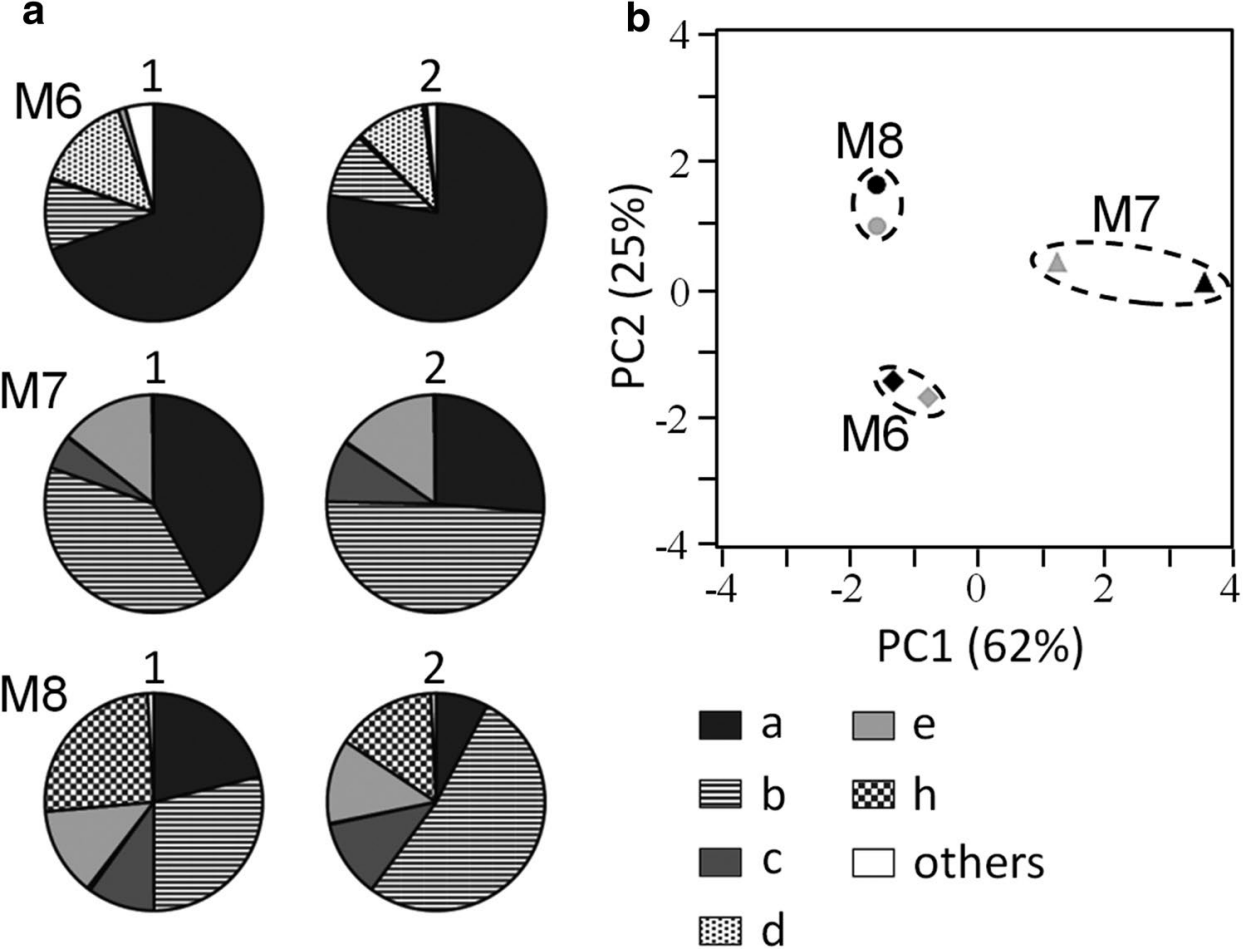
Temporal changes in chemical profiles of volatile compounds emitted from anal sac secretions of three cats **a** Pie charts of chemical profiles of 10 free fatty acids emitted from anal sac secretions of three cats (M6–M8). *a* Acetic acid, *b* propanoic acid, *c* 2-methylpropanoic acid, *d* butanoic acid, *e* 3-methylbutanoic acid, *h* 4-methylpentanoic acid. *Others* indicate a mixture of pentanoic acid, 3-methyl-pentanoic acid, hexanoic acid, and 4-methylhexanoic acid. *1* and *2* on the pie charts indicate first and second samples (obtained after 2 months from first sampling) of anal sac secretions. **b** PCA was carried out using GC–MS data of 10 free fatty acids emitted from anal sac secretions. *Black* and *gray* spots indicate the first and the second samples, respectively

We next examined whether there are fixed patterns in volatile chemical profiles of anal sac secretions in each cat. For this study, we collected the anal sac secretions of three male cats (M6–M8) at two points in time (day 1 and day 72), and analyzed the headspace gas of the secretions. Figure 2a shows pie charts of the chemical profiles of the free fatty acids emitted from the anal sac secretions of the three cats. The chemical composition of each free fatty acid was calculated by dividing the content of each free fatty acid by the total amounts of the 10 free fatty acids. Although the amounts of each free fatty acid were different between samples obtained on day 1 and day 72 in each cat, we can see the tendency that each cat had similar patterns at the two points in time. Thus, we next carried out PCA using the data set. PCA, in which the first two principal components together explained 87% of the total variance in the chemical profiles, shows that the chemical profiles of free fatty acids were highly similar between two anal sac secretion samples of each cat (Fig. 2b).

### Habituation–dishabituation tests in cats

**Fig. 3:**
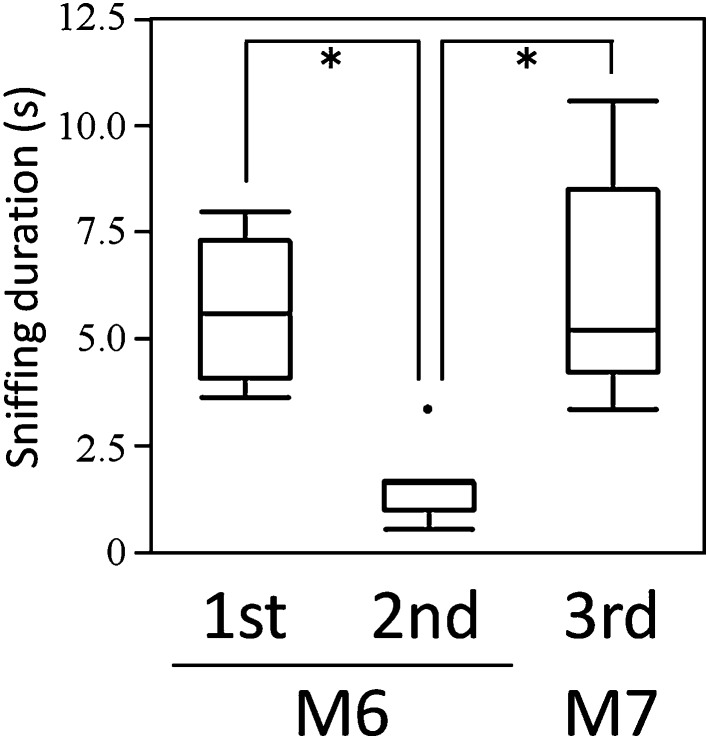
Olfactory habituation-dishabituation tests in cats Habituation–dishabituation tests were carried out in 5 male cats (M1–M5) using anal sac secretions obtained from another two male cats (M6 and M7). The first presentation of the M6 sample elicited sniffing that decreased at second presentation (habituation). Subsequent presentation of the M7 sample elicited significantly more sniffing than the second presentation (dishabituation). *Asterisks* indicate significant differences (*p* < 0.05)

We examined the ability of cats to discriminate between anal sac secretions in olfactory habituation–dishabituation tests. The test helps determine, using the indicator of sniffing duration, whether animals can habituate to a repeatedly presented odor and whether the animals demonstrate dishabituation when presented with a novel odor (Arbuckle et al. 2015). If we observe both a significant decrease of sniffing duration by repeated presentation of the same stimulus (habituation response) and a significant increase of sniffing duration by the presentation of the other stimulus (dishabituation response), the test determines that the animals can discriminate between two stimuli using olfaction. To avoid presentations of their own anal sac secretions to the five cats (M1–M5) used in the tests (because they may have remembered and habituated to their own secretions before the tests), we used anal sac secretions obtained from the other male cats (M6–M8). Sniffing duration was measured when the cats twitched their noses to sample the odors, which was observed when the cat’s nose was within 5 cm of the tube opening. In preliminary experiments using the anal sac secretion obtained from a male cat (M8), we confirmed that the cats remembered and habituated to the sample odor, shown by a significant decrease in sniffing duration with second presentations. However, more than three presentations could negatively affect the sniffing test; for example, the cats might avoid the test samples, or rest on the opposite side of the test chamber to that containing the sample. Therefore, in the habituation–dishabituation tests using anal sac secretions of two male cats (M6 and M7) we presented the M6 sample twice for the habituation trials, and then the M7 sample once. In the habituation trials, the sniffing duration for the M6 sample decreased significantly in second presentations in all five intact male cats (Fig. 3). Then, a significant increase in sniffing duration was observed in the cats that sniffed the M7 sample, indicating a dishabituation response. These results demonstrate that cats can distinguish individual differences in odors emitted from two anal sac secretions.

## Discussion

In this study, we found that a major driver of variation in the chemical composition of short-chain free fatty acids was individual differences among cats. These short-chain free fatty acids had already been identified in anal sac secretions from several other mammalian species such as mongooses, suggesting that these compounds are not chemical signals for species recognition in cats (Albone et al. 1974; Preti et al. 1976; Apps et al. 2012; Miyazaki et al. 2017b). Previous studies found that cat urine emits sulfur-containing volatile compounds, 3-mercapto-3-methylbutanol and 3-mercapto-3-methylbutyl formate, which are the major contributors to the specific sulfurous odor of cat urine (Joulain and Laurent 1989; Miyazaki et al. 2006). Felinine, a precursor of these compounds, has also never been identified from mammalian excretions and secretions other than felids (Hendriks et al. 1995). We did not identify these cat-specific compounds in the headspace gas of anal sac secretions. On the other hand, previous comparative studies have suggested that trimethylamine, with its distinctive fishy odor, is detectable in the anal sac secretions of dogs but not cats (Preti et al. 1976), but we detected trimethylamine as a minor compound in the anal sac secretions of cats, although the carbotrap adsorbent is suitable for the analysis of trimethylamine as compared to Tenax-TA (Krzymien and Elias 1990). There was no sex difference in the chemical profiles of the anal sac secretions in cats. In addition, our data strongly suggest that there are fixed patterns in volatile chemical profiles of anal sac secretions in each cat. These findings indicate that anal sac secretions convey information on individual cats rather than species or sex information.

We demonstrated that male cats can distinguish among the individual odors of anal sac secretions, from which they obtain scent information for recognition of individuals. Considering also the chemical profiles of anal sac secretions, we propose that cats utilize scent signals emitted from anal sac secretions of other male cats for recognition of individuals. In this study, however, we did not examine whether female cats can distinguish among the individual odors of anal sac secretions, as well as male cats. Because, to examine the olfactory abilities for recognition of individuals by habituation–dishabituation tests in five female cats, we would have had to obtain anal sac secretion samples from a further two female cats, but could not do it. Further behavioral bioassays using female cats will improve our understanding of recognition of individuals using anal sac secretions in domestic cats.

Previous studies have suggested that anal sac secretions are added to urine when cats spray for scent marking, or are deposited on the surface of feces during passage through the anal canal (Feldman 1994). However, in large felids such as lions, no anal sac secretions labeled with an inert dye were detectable in urinary or fecal markers (Asa 1993). Therefore, it is postulated that anal sac secretions are not mainly used for scent marking in cats. We are currently examining the relationship between anal sac secretions and the odor of the perianal area in cats. Cats prefer to sniff the perianal area of other cats when they encounter other individuals in a friendly mood (Crowell-Davis et al. 2004). Anal sac secretions might contribute to the odor of the perianal area and convey individual information in addition to visual information, regarding for example the face or body shape of the other cat. Such double-checking of individual identity using both visual and olfactory information may enable cats to distinguish among individuals, even if their appearances are similar due to close relatedness within a family.

Olfactory discrimination of the anal sac secretions between conspecific individuals has been demonstrated to be based on the differences in free fatty acid composition in mongooses (Gorman 1976). The bacterial action in the anal sac has been found to contribute to produce short-chain free fatty acids in red fox, lion, mongoose, and wolves (Albone et al. 1974; Gorman et al. 1974; Gosden and Ware 1976; Raymer et al. 1985). In healthy cats, a mixed bacterial population, dominated by Gram-positive cocci, Gram-negative cocci and rods, and occasional yeasts, has been identified from the anal sac secretions (Frankel et al. 2008). A recent study identified *Propionibacterium* (*Cutibacterium*) *avium* strain UCD-PD2, an anaerobic Gram-positive bacterium, from the anal sac secretion of a domestic cat, which may contribute to the odor of anal sac secretions (Dahms et al. 2017). These suggest that the bacterial flora present in anal sacs of domestic cats also contribute to distinctive individual odors by producing short-chain free fatty acids which the cats can discriminate.

In conclusion, we demonstrated that cats can distinguish individual differences in the odor of anal sac secretions by their olfaction. As in other carnivores, the major volatile compounds in the anal sac secretions are short-chain free fatty acids; the contents varied markedly among individuals, but not by sex. Based on our results, we postulate that short-chain free fatty acids emitted from anal sac secretions are key compounds for recognition of individuals by olfaction in cats.
